# The reporting of a *Bacillus anthracis* B-clade strain in South Africa after more than 20 years

**DOI:** 10.1186/s13104-018-3366-x

**Published:** 2018-05-02

**Authors:** K. E. Lekota, A. Hassim, P. Rogers, E. H. Dekker, R. Last, L. de Klerk-Lorist, H. van Heerden

**Affiliations:** 1 Department of Veterinary Tropical Diseases, Faculty of Veterinary Science, University of Pretoria, Onderstepoort, South Africa; 2 Provet Wildlife Services, Raptors Safari Junction, Main Road, Hoedspruit, South Africa; 3 State Veterinary Services, Department of Agriculture, Forestry and Fisheries, Skukuza, South Africa; 4 Vetdiagnostix–Veterinary Pathology Services, 257 Boshoff Street, Pietermaritzburg, South Africa; 5 College of Agriculture and Environmental Sciences, University of South Africa, Christiaan De Wet/Pioneer Dr., Florida, South Africa

**Keywords:** *Bacillus anthracis*, Whole genome sequencing (WGS), Single nucleotide polymorphisms (SNPs)

## Abstract

**Objectives:**

Anthrax is a disease with an age old history in Africa caused by the Gram-positive endospore forming soil bacterium *Bacillus anthracis.* Epizootics of wild ungulates occur annually in the enzootic region of Pafuri, Kruger National Park (KNP) in the Limpopo Province of South Africa. Rigorous routine surveillance and diagnostics in KNP, has not revealed these rare isolates since the 1990s, despite unabated annual outbreaks. In 2011 a cheetah was diagnosed as anthrax positive from a private game reserve in Limpopo Province and reported to State Veterinary Services for further investigation. Isolation, molecular diagnostics, whole genome sequencing and comparative genomics were carried out for *B. anthracis* KC2011.

**Results:**

Bacteriological and molecular diagnostics confirmed the isolate as *B. anthracis*. Subsequent typing and whole genome single nucleotide polymorphisms analysis indicated it clustered alongside *B. anthracis* SA A0091 in the B.Br.010 SNP branch. Unlike *B. anthracis* KrugerB strain, KC2011 strain has unique SNPs and represents a new branch in the B-clade. The isolation and genotypic characterisation of KC2011 demonstrates a gap in the reporting of anthrax outbreaks in the greater Limpopo province area. The identification of vulnerable and susceptible cheetah mortalities due to this strain has implications for conservation measures and disease control.

**Electronic supplementary material:**

The online version of this article (10.1186/s13104-018-3366-x) contains supplementary material, which is available to authorized users.

## Introduction

Anthrax is a zoonotic disease with an age old history in Africa [1], causing acute mortalities. It primarily affects ungulates with episodic spill over into humans and carnivores [2, 3]. The causative agent of this disease is the Gram positive, endospore forming, exotoxin producing soil bacterium *Bacillus anthracis* [4, 5]. Sporulation is triggered by nutrient scarcity, the presence of bicarbonate and oxygen exposure [6, 7]. The virulence factors of this bacterium are on the two plasmids, pXO1 (181 kilobases) and pXO2 (94 kilobases), encoding the toxin and capsule genes respectively [8, 9]. The toxin complex is composed of three components namely, lethal toxin (LF) made up of the protective (PA) and lethal factor (EF) [10, 11]. The capsule consists of a five gene operon (*capBCADE*) that produces a poly-gamma-d-glutamic acid (PGA) on the bacterial cell membrane that protects the vegetative cells from phagocytosis [12, 13].

Anthrax was widespread across southern Africa in the early 20th Century, until the development and South African state implementation of the Sterne vaccine in 1937 [14, 15]. While livestock cases have since been infrequent due to vaccination practices; outbreaks are still common amongst wildlife in endemic regions of South Africa and game reserves across the continent [2, 16]. Limpopo province covers 125,754 km^2^ bordering Botswana and Zimbabwe to the north. The northern half of the Kruger National Park (KNP) makes up the entire eastern border of the Limpopo Province bordering Mozambique. The anthrax endemic region in KNP falls between the Limpopo and Luvhuvhu rivers [19]. The distribution of outbreaks and the animal species affected by anthrax have been described extensively in KNP in the last 35 years [17–20]. The genetic population structure of *B. anthracis* consists of the global A, B and C clades [21]. The B clade isolates identified in South Africa were confined to the northern tip of KNP in Limpopo Province due to it being an ideal soil environment for spore persistence [17]. The A clade isolates, in contrast, have a wide distribution, predominating and prevailing over the rarer B clade isolates during outbreaks since the late 1980s.

Host species differ in their susceptibility to *B. anthracis.* Wild ungulates are more susceptible to the disease than carnivores [2, 3]. Even amongst herbivores, roan antelope (*Hippotragus equinus*) are far more susceptible to anthrax than other antelope species [20]. Similarly, cheetahs (*Acinonyx jubatus*) are more susceptible to the toxins produced by the bacterium than other carnivorous felids and canids [22–24]. It is hypothesised that this could be due to the immune response dictated by the monomorphic genetic structure of southern African cheetahs [25] or by the feeding habits of free roaming cheetah [22]. In either event, it is of importance to cheetah conservation practices since these felids are considered vulnerable (bordering on endangered) [26].

**Fig. 1:**
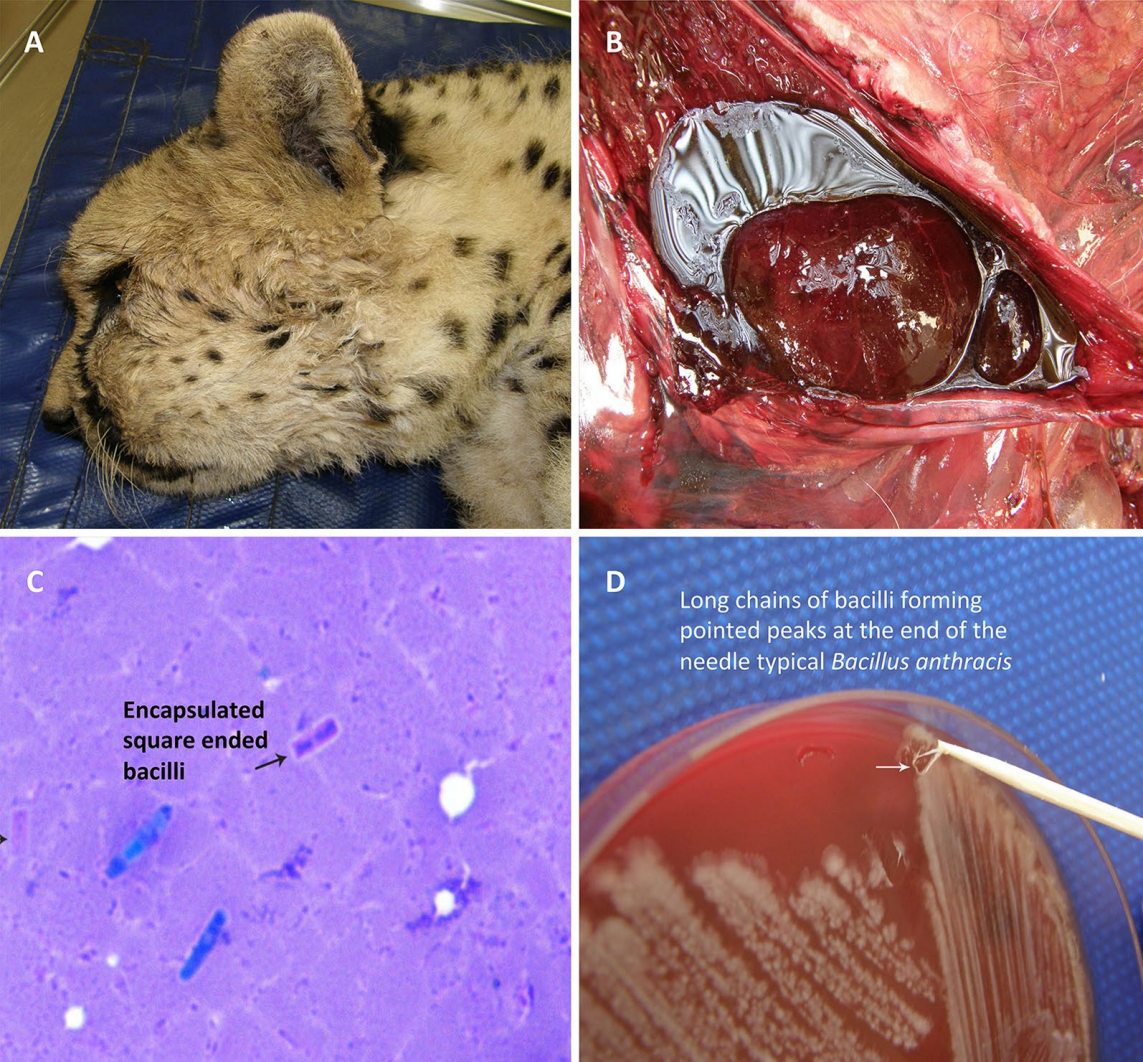
**A** Cheetah (*Acinonyx jubatus*) demonstrating facial oedema in reaction to *Bacillus anthracis* toxins. **B** Dark un-clotted blood surrounding the kidney of a cheetah indicating infection by *B. anthracis.***C** Impression smear of oedematous fluid from cheetah with typical square ended *B. anthracis* bacilli (purple bacilli indicated by the arrow). **D** Bacteriologic diagnostics on 5% sheep blood agar demonstrating typical *B. anthracis* colony morphology and structure

This status has led to more stringent investigations of cheetah mortalities in both captive and free roaming animals. In 2011 a cheetah mortality from a private game reserve in Limpopo Province was investigated by the local veterinarian. The cheetah was observed to have substantial facial oedema with unclotted blood around the kidneys (Fig. 1A, B). Biological samples from the cheetah were submitted for bacteriologic diagnostics (Fig. 1C, D). Colony morphology indicative of *B. anthracis* then confirmed the initial diagnosis (Fig. 1D). The isolate was then submitted by Skukuza State Veterinary Services for further analysis on behest of the Anthrax Advisory Committee.

## Main text

### Methods

#### Isolation and identification

A Giemsa smear stain from the oedema fluid was performed for identification of *B. anthracis*. Pure culture isolation of KC2011 isolate was obtained by culturing biological samples directly onto 5% sheep blood agar after 24 h incubation at 37 °C characteristics including colony and cell morphology, gamma-phage and penicillin sensitivity were evaluated as described in OIE [3]. Capsule formation was observed by visualization using light microscopy of Giemsa stained fluid and colonies.

#### DNA extractions and qPCR

Genomic extraction of *B. anthracis* KC2011 was carried out using a DNA Blood Mini Kit (Qiagen, Germany) according to manufacturer’s instructions. The DNA was quantified using Qubit™ fluorometric quantitation (Invitrogen™, USA). Quality of the extracted DNA was visualized on 0.8% agarose gel electrophoresis. The diagnostic qPCR for *B. anthracis* was performed with 2.5 µL DNA in 1× FastStart™ Taq DNA Polymerase mastermix (Roche^®^, Germany) and 0.5 μM of each primer small acid soluble proteins (SASP), *Bacillus anthracis* protective antigen (BAPA), and capsule region (*cap*C) along with 0.2 μM of probe for each chromosomal and plasmid target pairs with fluorescein on the one and LCRed640 on the other (Tib MolBiol GmbH, Germany) in a final volume of 20 µL as described in Turnbull [3]. The PCR conditions on a LightCycler™ Nano (Roche^®^, Germany) were used as described in OIE [3, 27].

#### Melt-MAMA assay

The PCR single nucleotide polymorphism (SNP) assay was performed for the oligonucleotide markers A/B.Br.001, B.Br.003, B.Br.004, B.Br.002, B.Br.001, A.Br.007 and A.Br.002 as described in Birdsell et al. [28]. The reaction included 2.5 µL DNA diluted in 1× FastStart DNA Green Master (Roche^®^, Germany) with an ancestral forward and a derived forward SNP target primer (GC-clamp: no-GC-clamp) and a common reverse primer with a starting concentration of 0.2 µM depending on the ratio indicated which allowed for separation of melt peaks by at least 5 °C. Thermocycling parameters on the LightCycler™ Nano (Roche^®^, Germany) were conducted as described in Birdsell et al. [28].

#### Whole genome sequencing and Bioinformatics analysis

Sequence libraries of the DNA isolate was generated using the Nextera DNA Sample Prep Kit (Illumina, USA) protocol. Sequence reads of paired end library was performed on a HiSeq 2500 sequencer (Illumina, USA). Quality of the genome sequenced reads were assessed using FastQC software 0:10.1 [29]. Trimmomatic [30] was used to remove the ambiguous nucleotide reads. De novo assembly of the *B. anthracis* KC2011 was carried out using the CLC Genomics Workbench version 7.5 (CLC, Denmark). The assembled contigs were aligned with BLASTn [31] using *B. anthracis* Ames ancestor (GenBank: AE017334.2, AE017336.2, and AE017336.2) as a reference. Mauve tool [32] was used to align and order the assembled contigs using *B. anthracis* Ames ancestor as a reference. The genome was annotated using PGAAP at NCBI [33].

The trimmed reads of *B. anthracis* KC2011 were aligned to *B. anthracis* Ames ancestor using the Burrows-Wheeler Aligner (BWA) [34]. SAMtools [35] was used to sort and index the aligned sequenced reads. Unified genotyper in GATK [36] was used to call for SNPs. In order to construct WGS- SNPs tree, complete and draft genomes from different clades of *B. anthracis* available in NCBI Genbank (http://www.ncbi.nom.nih.gov) were included in this study (Additional file 1: Table S1). SNPs positioning sets were deducted from the aligned genomes of *B. anthracis* Ames ancestor using molecular evolutionary genetics analysis (MEGA) 7 [37]. SNPs with informative sites (core SNPs) in all genome sequences were used for the phylogenetic tree construction using MEGA 7 tool [37].

#### Genbank submission

The genome sequence of *B. anthracis* KC2011 was deposited in the Genbank genome database under the accession number: NJGK00000000.

### Results

#### Isolation and identification

A Giemsa stained impression smear from the oedema fluid revealed encapsulated, square ended bacilli pathognomonic for *B. anthracis* (Fig. 1C). The microbiological characteristics confirmed the bacterium to be *B. anthracis* on blood smear with typical *B. anthracis* colony morphology and structure on 5% sheep blood agar [38] (Fig. 1D).

#### qPCR and Melt-MAMA analysis

The *B. anthracis* KC2011 isolate was confirmed positive for the presence of *B. anthracis* BAPA, SASP and *capC*. *B. anthracis* KC2011 was further typed using Melt-MAMA and amplified the derived markers for A/B.Br.001, B.Br.003 and B.Br.002, whilst amplifying the B.Br.001 ancestral marker. This SNP profile indicated KC2011 to be a B clade (B.Br.001/002), but not the rare KrugerB sub-clade.

#### Genome assembly and phylogeny

De novo assembly was carried out which resulted in 45 contigs (Table 1). The total genome contigs contributed to a 5.42 MB, with a GC content of 35%. A total of 6 047 coding sequences (CDSs) was determined in the KC2011 strain (Table 1). Comparative genome alignment of the *B. anthracis* KC2011 genome with Ames ancestor revealed no evidence of novel genes (Additional file 3: Fig. S1). The genome coverage of *B. anthracis* KC2011 was 99% covered to *B. anthracis* Ames ancestor (Additional file 1: Table S1; Additional file 2: Table S2). High copy number of pXO1 was observed represented by 725 coverage (725×).

**Table 1:** Genome features of the *Bacillus anthracis* KC2011

Features	*Bacillus anthracis* KC2011 genome
Domain	Bacteria
Genome coverage (X)	358
Avg. length after trim	118
Genome size (bp)	5 421 567
Number of contigs	45
Maximum length	500 750
Minimum length	9000
G+C content (%)	35.2
Genes (total)	6 047
CDS (coding)	5964
Genes (RNA)	83
Complete rRNAS	4, 1 (5S, 23S)
Number of tRNA	73
Number of ncRNAs	5
Pseudo genes (total)	322

**Fig. 2:**
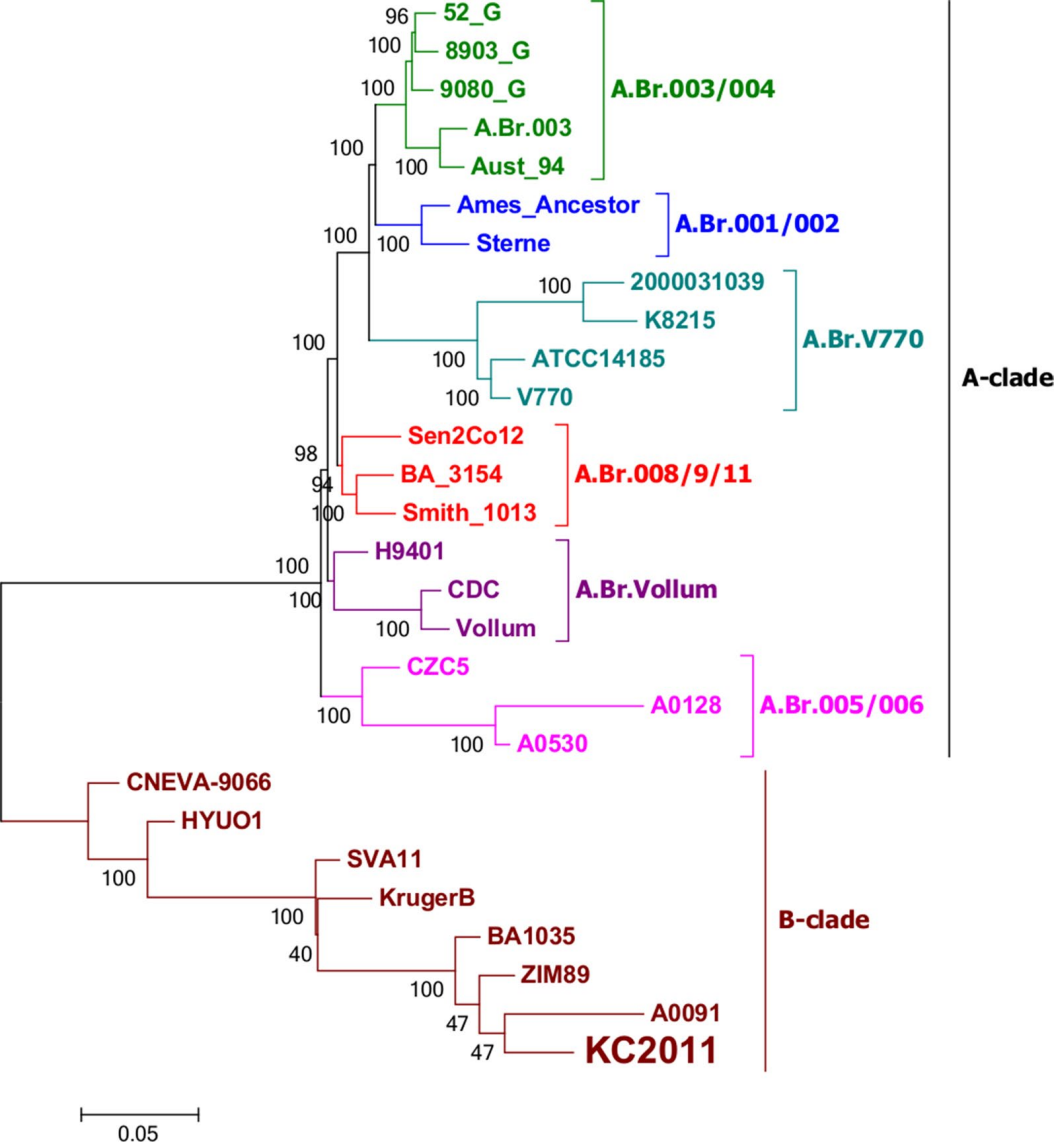
Whole genome SNP phylogeny of the global *Bacillus anthracis* strains indicating the placement of KC2011 strain. Maximum likelihood was constructed using 3 247 parsimony informative SNPs. The rare *B. anthracis* KC2011 (indicated with higher font) grouped in the B-clade separately from KrugerB strain

A high resolution tree of global *B. anthracis* strains was constructed to visualise the SNP grouping of the *B. anthracis* KC2011 (Fig. 2). This yielded 3 247 parsimony informative SNPs that were used to construct the phylogram. The *B. anthracis* KC2011 grouped in the B-clade separate from the KrugerB strain. The closest related branch was SA *B. anthracis* A0091.

### Discussion

Strain KC2011 is the first *B. anthracis* identified as a B-clade lineage bacterium in over 20 years in South Africa and represents a new subclade in the B-clade. About 90% of the anthrax outbreaks reported world-wide arise from the A-clade, while less than 10% have been reported from the B-clade (KrugerB subclade) [21]. The majority of the *B. anthracis* strains isolated from the northern part of KNP from 1970 to 1981 grouped in the B-clade (KrugerB subclade) but this clade became rare since the 1990s [17]. Only one B strain reported between 1982 and 1997 [19] to the extent that it was no longer isolated from 1997 until this report in South Africa.

No significant anthrax outbreaks were noted outside the endemic regions of KNP (northern part of KNP) in 2011 according to state veterinary surveillance data. The isolate KC2011 was only identified because of the delicate conservation status of cheetah in Africa and because it was within the context of the broader Limpopo province area (i.e. outside KNP). Cheetahs are especially susceptible to the *B. anthracis* exotoxins, however, they do respond well to vaccination with the Sterne vaccine [25]. This study highlighted the importance of vaccinating cheetah, where logistically possible, in conservancies across Limpopo province.

The phylogenetic structure of *B. anthracis* determined in this study (Fig. 2) is similar to other reported WGS-SNP trees [39–42]. Past B-clade (1965–1990) isolates from the State Veterinary archival collection have all clustered in the KrugerB lineage. WGS-SNP analysis defined KC2011 strain grouping as a distinctive sub-clade within southern Africa B.Br.010.

Although, distant in terms of number of SNPs, the isolate A0091 and KC2011 belong to a common albeit broader lineage. The isolate A0091 is recorded as being from South Africa circa 1939, but has no other information to link it to KNP or Limpopo Province. There is also a dearth of information from the countries (Zimbabwe and Mozambique) bordering the South African province across the Limpopo River. Further surveillance of Limpopo Province is required to reveal whether deposits of B-clade isolates are subsisting outside KNP or whether this was an incidental mortality due to circumstances outside the realm of current monitoring criteria. Surveillance in the non-endemic anthrax regions is difficult as there are private game reserves and rural livestock farmers bordering KNP. Moreover the rural subsistence farmers (predominantly cattle farmers) are less likely to report mortalities and/or vaccinate their livestock thus complicating disease monitoring and control. This report of the *B. anthracis* KC2011 isolated outside the endemic region highlights the necessity of disease surveillance in non-endemic regions.

### Conclusion

The identification of KC2011 is significant in that mortalities linked to the B-clade have diminished over several decades and therefore was estimated to have disappeared from the local environment. This suggests a possible gap in surveillance of carcasses submitted for anthrax diagnostics in the Limpopo province especially in the non-endemic regions. It also has implications for the inclusion of the Sterne vaccine as part of the conservation measures to be included for cheetah in sanctuaries and private game reserves across the Limpopo Province in South Africa.

## Limitations

The tree topology of the southern African strains was not well characterised due to the limited number of genomes available. Branch lengths vary according to inclusion of number and variety genomes in the data sets, as this explicates the informative SNPs used to construct the tree. Isolate KC2011 will thus be the basis for further typing of *B. anthracis* strains for southern Africa.

## Additional files

**Additional file 1: Table S1.** Whole genomes of *Bacillus anthracis* retrieved from public database used in this study.

**Additional file 2: Table S2.** Genome alignment of the *Bacillus anthracis* KC2011 to the *B. anthracis* Ames ancestor reference.

**Additional file 3: Fig. S1.** Alignment of *Bacillus anthracis* Ames ancestor genome with *B. anthracis* KC2011. Each colour block indicates homologous regions of the genome sequences. White areas in colour blocks indicate possible nucleotide variation absence or presence within the compared genomes.

## Abbreviations

PGA: poly-gamma-d-glutamic acid
LF: lethal toxin
PA: protective
EF: lethal factor
KNP: Kruger National Park
WGS: whole genome sequencing
SNP: single nucleotide polymorphism
Melt-MAMA: melting mismatch amplification mutation analysis
CDSs: coding sequences
