# Supplementary material for: The reporting of a Bacillus anthracis B-clade strain in South Africa after more than 20 years
Source: BMC Res Notes. 2018 May 2;11:264. doi: 10.1186/s13104-018-3366-x (PMC5930959; doi:10.1186/s13104-018-3366-x)
Supplement: Supplementary file 1 — Additional file 1: Table S1. Whole genomes of Bacillus anthracis retrieved from public database used in this study. [file 13104_2018_3366_MOESM1_ESM.docx]

Additional file 1: Table S1. Whole genomes of *Bacillus anthracis* retrieved from public database used in this study.

| **Strain** | **Country** | **canSNP** | **Accession number** |
| --- | --- | --- | --- |
| Ames ancestor | USA | A.Br.Ames | NC_007530.2 |
| Sterne | South Africa | A.Br.001/002 | AE017225.1 |
| 52_G | Georgia | A.Br.Australia94 | AZUF00000000.1 |
| 8903_G | Georgia | A.Br.Australia94 | AZUD00000000.1 |
| 9080_G | Georgia | A.Br.Australia94 | AZUE00000000.1 |
| V770 -NP-1R | USA | A.Br.003/004 | AZQO00000000 |
| 2000031039 | USA | A.Br.003/004 | JSZR01000000.1 |
| A1039 | Bolivia | A.Br.003/004 | LAKZ01000000.1 |
| K8215 | Argentina | A.Br.003/004 | LGIG01000000.1 |
| ATCC14185 | Israel | A.Br.003/004 | AZQO00000000.1 |
| A.Br003 | Scotland | A.Br.Australia94 | JMPV00000000.1 |
| Aust94 | Australia | A.Br.Australia94 | GCA_000167335.1 |
| BA_3154 | Bulgaria | A.Br.009/011 | ANFF00000000.1 |
| Smith_1013 | Unknown | A.Br.009/011 | JNOD00000000.1 |
| CDC684 | USA | A.Br.Vollum | NC_012581.1 |
| A0530 | Botswana | A.Br.005/006 | SRR2968170, PRJNA302749, SAMN04283802 |
| A0128 | South Africa | A.Br.005/006 | SRR2968156, PRJNA302749, SAMN04283804 |
| K3 | South Africa | A.Br.005/006 | CP009329.1, CP009330.1, CP009331.1 |
| CZC5 | Zambia | A.Br.005/006 | BAVT00000000.1 |
| H9401 | Korea | A.Br.H9401 | NC_017729.1 |
| Vollum | UK | A.Br.Vollum | AAEP00000000.1 |
| Sen2Co12 | Africa | A.Br.0011/009 | CAVC000000000.1 |
| CNEVA-9066 | France | B.Br.CNEVA | NZ_AAEN00000000.1 |
| KrugerB | South Africa | B.Br.Kruger | AAEQ00000000.1 |
| HYU01 | South Korea | B.Br.001/002 | CP008846 |
| SVA11 | Sweden | B.Br.001/002 | CP006742.1 |
| BA1035 | South Africa | B.Br.001/002 | CP009698.1, CP009699.1, CP009700.1 |
| A0091 | South Africa | B.Br.001/002 | SRR2968134, PRJNA302749, SAMN04283795 |
