# Supplementary material for: The reporting of a Bacillus anthracis B-clade strain in South Africa after more than 20 years
Source: BMC Res Notes. 2018 May 2;11:264. doi: 10.1186/s13104-018-3366-x (PMC5930959; doi:10.1186/s13104-018-3366-x)
Supplement: Supplementary file 2 — Additional file 2: Table S2. Genome alignment of the Bacillus anthracis KC2011 to the B. anthracis Ames ancestor reference. [file 13104_2018_3366_MOESM2_ESM.docx]

Additional file 2: Table S2. Genome alignment of the *Bacillus anthracis* KC2011 to the *B. anthracis* Ames ancestor reference.

|  | **Consensus length** | **Total read count (pairs)** | **Coverage**  **(x)** | **Reference length** |
| --- | --- | --- | --- | --- |
| Chromosome | 5 188 197 | 14 314 894 | 324 | 5 227 419 |
| pXO1 | 181 654 | 11 142 73 | 725 | 181 677 |
| pXO2 | 94 777 | 3 840 30 | 479 | 94 830 |
