# Supplementary figures and images for: The reporting of a Bacillus anthracis B-clade strain in South Africa after more than 20 years
Source: BMC Res Notes. 2018 May 2;11:264. doi: 10.1186/s13104-018-3366-x (PMC5930959; doi:10.1186/s13104-018-3366-x)

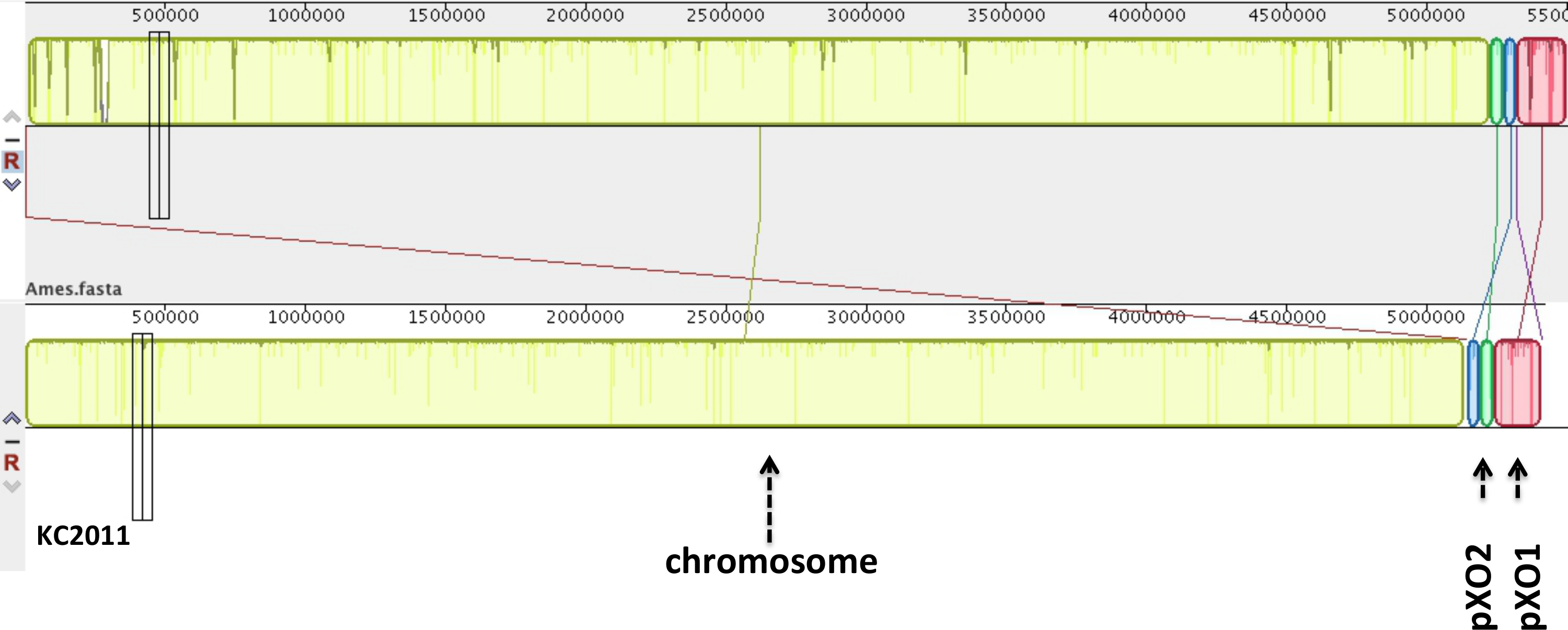

Supplement: Supplementary file 3 — Additional file 3: Fig. S1. Alignment of Bacillus anthracis Ames ancestor genome with B. anthracis KC2011. Each colour block indicates homologous regions of the genome sequences. White areas in colour blocks indicate possible nucleotide variation absence or presence within the compared genomes. [file 13104_2018_3366_MOESM3_ESM.tiff]
